# ALK1 signalling analysis identifies angiogenesis related genes and reveals disparity between TGF-β and constitutively active receptor induced gene expression

**DOI:** 10.1186/1471-2261-6-13

**Published:** 2006-04-04

**Authors:** Andreas Lux, Fiona Salway, Holly K Dressman, Gabriele Kröner-Lux, Mathias Hafner, Philip JR Day, Douglas A Marchuk, John Garland

**Affiliations:** 1 University Hospital Mannheim, 68167 Mannheim, University of Applied Sciences Mannheim, Windeckstr. 110, 68163 Mannheim, Germany; 2 Institute of Molecular and Cell Biology, University of Applied Sciences Mannheim, Windeckstr. 110, 68163 Mannheim, Germany; 3 Centre for Integrated Genomic Medical Research, University of Manchester, Manchester, M13 9PT, UK; 4 Department of Molecular Genetics and Microbiology, DUMC, Durham, NC 27710, USA; 5 Duke Institute for Genome Sciences and Policy, DUMC, Durham, NC 27710, USA; 6 PROGEN Biotechnik GmbH, Maasstr. 30, 69123 Heidelberg, Germany; 7 Manchester Cardiovascular Research Group, University of Manchester, Department of Medicine, M13 9WL, UK

## Abstract

**Background:**

TGF-β1 is an important angiogenic factor involved in the different aspects of angiogenesis and vessel maintenance. TGF-β signalling is mediated by the TβRII/ALK5 receptor complex activating the Smad2/Smad3 pathway. In endothelial cells TGF-β utilizes a second type I receptor, ALK1, activating the Smad1/Smad5 pathway. Consequently, a perturbance of ALK1, ALK5 or TβRII activity leads to vascular defects. Mutations in *ALK1 *cause the vascular disorder hereditary hemorrhagic telangiectasia (HHT).

**Methods:**

The identification of ALK1 and not ALK5 regulated genes in endothelial cells, might help to better understand the development of HHT. Therefore, the human microvascular endothelial cell line HMEC-1 was infected with a recombinant constitutively active ALK1 adenovirus, and gene expression was studied by using gene arrays and quantitative real-time PCR analysis.

**Results:**

After 24 hours, 34 genes were identified to be up-regulated by ALK1 signalling. Analysing ALK1 regulated gene expression after 4 hours revealed 13 genes to be up- and 2 to be down-regulated. Several of these genes, including *IL-8*, *ET-1*, *ID1*, *HPTPη *and *TEAD4 *are reported to be involved in angiogenesis. Evaluation of ALK1 regulated gene expression in different human endothelial cell types was not in complete agreement. Further on, disparity between constitutively active ALK1 and TGF-β1 induced gene expression in HMEC-1 cells and primary HUVECs was observed.

**Conclusion:**

Gene array analysis identified 49 genes to be regulated by ALK1 signalling and at least 14 genes are reported to be involved in angiogenesis. There was substantial agreement between the gene array and quantitative real-time PCR data. The angiogenesis related genes might be potential HHT modifier genes. In addition, the results suggest endothelial cell type specific ALK1 and TGF-β signalling.

## Background

Vascular development and homeostasis are regulated by a number of cytokines including members of the transforming growth factor-beta (TGF-β) superfamily that resemble a group of structurally related secreted polypeptides that regulate numerous cellular activities including proliferation, differentiation, migration, extracellular matrix deposition and apoptosis [1,2]. This family consists of over 35 cytokines that include TGF-β1, -β2 and -β3, as well as activins, inhibins, nodals and the large group of bone morphogenetic proteins (BMPs). All have crucial roles in development and tissue homeostasis and their importance is further demonstrated by their involvement in different diseases [1,3].

Signalling is mediated by a class of single transmembrane domain serine/threonine kinase receptors, types -I and -II, that initiate phosphorylation of co-transcription factors of the Smad protein family [2,4]. There are five type II receptors and 7 type I receptors designated as activin receptor-like kinases (ALKs), ALK1-7. Ligand binding induces complex formation between type I and type II receptors, upon which the constitutively active kinase of the type II receptor phosphorylates the type I receptor in its so called 'GS' domain. Activated type I receptor in turn phosphorylates receptor-regulated Smads (R-Smads; Smad-1, -2, -3, -5 and -8), which bind to the Smad4 protein, translocate to the nucleus and regulate gene expression in concert with other transcription factors. A third class of Smads, the inhibitory Smads (I-Smads; Smad-6 and -7), oppose the signalling activity of R-Smads and Smad4 by different mechanisms. Each TGF-β family member binds to a characteristic set of type I and II receptors and based on this combination activates a specific R-Smad.

In angiogenesis the formation of new blood vessels by de-novo capillary development from pre-existing vascular endothelium, vessel assembly, maturation and remodelling is based on a finely balanced series of events in which TGF-β plays a pivotal role, both as a pro-angiogenic (activation phase) as well as an anti-angiogenic (resolution-maintenance phase) cytokine [5,6]. This bi-phasic activity is dose-dependent [7,8]. Many angiogenic disorders most likely result from an unbalanced activity or loss of different angiogenic factors. Hereditary hemorrhagic telangiectasia (HHT) is characterized by telangiectases and arteriovenous malformations (AVMs) typically found in the skin and mucocutaneous tissues [9-11]. Telangiectases and AVMs show abnormal connection between arteries and veins that is devoid of intervening capillaries and has a more vein-like phenotype [12]. Previous studies have shown that HHT is caused by mutations in either *endoglin *(*CD105*) or *ALK1 *[13,14]. More recently, mutations in *Smad4 *were reported to cause a syndrome consisting of both juvenile polyposis and hereditary haemorrhagic telangiectasia phenotypes [15].

Endoglin binds TGF-β1 and -β3 isoforms that requires presence of the TGF-β type II receptor [16,17]. ALK1, a type I receptor for TGF-β1 and -β3 [6,18], activates the BMP signalling pathway by phosphorylating Smad1/5 [19,20]. Both endoglin and ALK1 are predominantly expressed by endothelial cells. Studies with mice have shown that a homozygous knock-out of either endoglin or ALK1 is embryonically lethal due to vascular defects [21-25]. Heterozygous endoglin as well as ALK1 mice are viable, but a percentage of those develop a phenotype similar to that observed in HHT patients [25,26].

Recent studies in endothelial cells have shown that TGF-β signals through ALK1, activating the Smad1/5 pathway, and through ALK5, activating the Smad2/3 pathway [6]. These two pathways have in a dose-dependent manner opposing effects on endothelial cell behaviour: ALK1 promotes cell proliferation and migration, whereas ALK5 inhibits both processes. Low TGF-β concentrations induce ALK1 signalling, which needs and is promoted by endoglin [27,28] and in addition requires the presence of ALK5 [29]. In contrast, high TGF-β concentrations activate the ALK5 pathway.

The lethality of endoglin or ALK1 deletion in mice points to essential roles for both in normal and aberrant angiogenesis but their exact roles are still largely unknown. To understand the function of endoglin and ALK1 in these processes requires elucidation of their activities in cell adhesion, migration, and importantly gene regulation. Gene arrays can simultaneously examine many hundreds or thousands of genes but suffer from being only semi-quantitative. Also, many changes are difficult to decipher due to high background that makes interpretation uncertain. Quantitative real-time (qRT)-PCR sensitively detects quantitative changes in mRNA but cannot process similarly large numbers of genes

In order to identify target genes specific to ALK1 signalling, we over-expressed ALK1 in the human microvascular endothelial cell line HMEC-1 using recombinant adenovirus carrying a constitutively active form of ALK1, thus masking ALK5 responses. We then compared gene array hybridisation data representing 5,600 human full-length genes, with semi-quantitative and qRT-PCR analyses of transcripts identified as altered by the Affymetrix GeneChip™ arrays. We found 34 genes to be up-regulated by constitutively active ALK1 signalling after 24 hours and 15 genes being dys-regulated (13 up- and 2 down-regulated) after 4 hours. Consistent with the vascular disorder HHT, at least 14 of the ALK1 response genes are reported to be involved in angiogenesis, vascular disorders or the homeostasis of the vascular system. We found substantial agreement between the gene array and qRT-PCR data, but there were a number of disparate results regarding TGF-β induced signalling and constitutively active receptor signalling depending on the endothelial cell type.

## Methods

### Cell culture and growth factor

HMEC-1 cell line (derived from human dermal microvascular endothelial cells) [30] was cultured in MCDB-131 containing 15% human serum, penicillin (100 U/ml), and streptomycin (100 μg/ml). ECRF24 cell line (derived from human umbilical vein endothelial cells) [31] was cultured in equal volumes of M199 and RPMI 1640 medium containing 2 mM glutamine, penicillin (100 U/ml), streptomycin (100 μg/ml), G418 (100 μg/ml), and 20% human serum. Primary human umbilical cord vein endothelial cells (HUVECs) were cultured in M199 glutamax containing HEPES (16 mM), heparin (6.4 U/ml), gentamicin (80 μg/ml), PD-ECGF (20 μg/ml), 10% foetal calf serum (FCS) and 10% human serum. Cre8 cells and HEK 293A cells were cultured in Dulbecco's modified Eagle's medium with 10% FCS, penicillin (100 U/ml), and streptomycin (100 μg/ml). All cells were cultured at 37°C in a humidified atmosphere of air containing 5% CO_2_. Media, media supplements and FCS were obtained from PAA Laboratories (Germany) and Invitrogen (Germany). TGF-β1 and PD-ECGF were obtained from R&D Systems (Wiesbaden, Germany).

### Recombinant adenovirus

The cDNA for constitutively active ALK1 (ALK1^QD^-HA, C-terminal HA tagged) was subcloned into the HindIII/BamHI sites of the Adlox vector, which possesses one loxP site, a left inverted terminal repeat followed by a packaging sequence (Ø), the human cytomegalovirus immediate-early promoter, a multiple cloning site, and a right inverted terminal repeat. The Adlox vector serves as the shuttle vector for generating recombinant adenovirus via the Cre-lox system [32]. Using CaPO_4_, Cre8 cells were co-transfected with the AdLox-ALK1^QD ^plasmid DNA (2.1 μg) and DNA (2.1 μg) of the E1-deleted φ5 virus (adenovirus type 5 engineered with two lox-P sites flanking the packaging site), creating the AdALK1^QD ^virus. For control infections virus containing no insert (AdCMV3) was created in addition to a virus expressing the bacterial β-galactosidase gene named AdLacZ. Following amplification of the recombinant virus in Cre8 cells, a large-scale virus preparation was made from infected HEK 293A cells. The cells were harvested 2 days after infection, sedimented (1000 × g, 10 min) and the cell pellet was resuspended in 10 ml of OPTI-MEM (Gibco-BRL), freeze-thawed three times, and resedimented. The resulting lysate (~10 ml) was loaded onto a two-step CsCl gradient (10 ml of CsCl at 1.4 g/cm^3 ^overlaid with 10 ml of CsCl at 1.2 g/cm^3^, and the gradient was centrifuged (20,000 rpm, 2 hr, 15°C, SW28 rotor). Viral particles in the 1.3 g/cm^3 ^region were harvested and the volume was brought to 21 ml with a solution of 1.3 g/cm^3 ^CsCl. In a tube the viral solution was overlaid onto a cushion of 1.5 ml of 1.5 g/cm^3 ^CsCl solution, and centrifuged (40,000 rpm, 12–16 hr, 15°C in an SW41 rotor). The resulting viral band (1.5–2.5 ml) was harvested and dialyzed at 4°C three times against 1 litre of buffer using a Slide-A-Lyzer (Pierce Chemical, Indianapolis, IN) and storage buffer (10 mM Tris-HCl, pH 7.4, 0.9% NaCl, 1 mM MgCl_2_, 1 mM CaCl_2_, with 10% glycerol in the last change) then aliquoted and stored at -80°C. Stock concentrations (viral particles [vp] per ml) were calculated from OD_260 _× dilution × 1.1 × 10^12^, with OD_260/280 _of 1.2–1.3.

### Viral infection

In order to test whether HMEC-1 cells can be infected by adenovirus, cells were incubated with AdLacZ at different multiplicity of infection (MOIs). An MOI of 50 was established to be optimal for 100% infection efficiency demonstrated by X-Gal staining (data not shown). Further on, an MOI of 50 displayed no effect on cell growth or morphology when tested for AdLacZ, AdCMV3 and more important for AdALK1^QD^, whereas MOIs of >100 affected cellular growth and induced partial cell death for all three tested recombinant adenoviruses. Once optimal MOI was established endothelial cells were seeded into a 100 mm dish at a density of ~4 × 10^6 ^cells. The next day cells were infected with an MOI of ~50 with the indicated recombinant adenovirus. In brief, medium was aspirated and exchanged for medium with no serum containing the virus. After 30 min of incubation the medium was aspirated and exchanged for fresh medium containing serum. Cells were then further incubated for the indicated time.

### RNA isolation and sample preparation for GeneChip™ hybridization

Total RNA from cells either infected with AdCMV3 or AdALK1^QD ^was isolated with the RNeasy Mini Kit (Qiagen) according to the manufacturer's instruction. Hybridization targets were prepared from 10 μg of total RNA according to standard Affymetrix™ protocols (see Affymetrix™ webpage [33]). Briefly, first strand cDNA synthesis was generated using a T7-linked oligo-dT primer, followed by second strand synthesis. An *in vitro *transcription reaction was performed to generate cRNA containing biotinylated UTP and CTP, which subsequently was fragmented chemically at 95°C for 35 min. The fragmented, biotinylated cRNA was hybridized in MES buffer (2-[N-morpholino]ethansulfonic acid) containing 0.5 mg/ml acetylated bovine serum albumin to the Affymetrix Human 6800 GeneChip™ at 45°C for 16 hr, according to the Affymetrix protocol. Arrays were washed and stained with streptavidin-phycoerythrin (SAPE, Molecular Probes). Signal amplification was performed using a biotinylated anti-streptavidin antibody (Vector Laboratories, Burlingame, CA) at 3 μg/ml, followed by a second staining with SAPE. Normal goat IgG (2 mg/ml) was used as the blocking agent.

Measurement scans were performed using an Affymetrix GeneChip™ confocal scanner and the MAS 4.0 average differences were computed. Scaling factors were determined for each hybridization based on a target intensity of 500. Scaled average difference values were used in generating comparison analysis in the Affymetrix MAS 4.0 software. Briefly, in the Affymetrix MAS 4.0 comparison analysis, one array is designated as the baseline (reference sample) and the other as an experiment (test sample). The analysis compares the difference values (perfect match-mismatch) of each probe pair in the baseline array (mock infected cells) to its equivalent on the experiment array (AdALK1^QD ^infected cells) and based on this a fold change was determined. Further explanation of the comparison analysis can be found at the Affymetrix™ webpage [33]. All GeneChip™ raw data generated from this study can be seen online [34] (24 hour experiments: 153_22hrCMV [reference sample] versus 154_22hrALK1 [test sample], 155_22hrCMV [reference sample] versus 156_22hrALK1 [test sample], 91_22hrCMV [reference sample] versus 92_22hrALK1 [test sample]; 4 hour experiments: 66_4hrCMV [reference sample] versus 63_4hrALK1 [test sample], 89_4hrCMV [reference sample] versus 90_4hrALK1 [test sample]).

### RNA isolation and semi quantitative RT-PCR

Total RNA from AdCMV3, AdLacZ or AdALK1^QD ^infected or non-infected cells and total RNA from TGF-β treated or non-treated cells was isolated with the RNeasy Mini Kit (Qiagen) according to the manufacturer's instruction. For further semi quantitative RT-PCR (sqRT-PCR) or qRT-PCR 2 μg of total RNA was reverse transcribed by random priming with hexamers and Oligo-dT primining (T_18_N) using the Omniscript RT Kit (Qiagen) according to the manufacturer's instruction. The resulted single strand cDNA was stored in a 50 μl volume at -20°C until use.

For sqRT-PCR 1 μl of the single strand cDNA was used in a 20 μl PCR reaction mixture containing 10 pmol of a gene specific primer pair (see Table 1), 1x PCR buffer (Invitrogen) containing 2.5 mM MgCl_2_, 200 μm dNTPs, 1 Unit Platinum Taq (Invitrogen). PCR conditions were as following: initial denaturing at 95°C for 5 min, 30 cycles of 30 sec denaturing at 95°C, 30 sec primer annealing at primer specific temperature, 30 sec elongation at 72°C. After 30 cycles final elongation step for 5 min. Equal amounts of PCR products were separated on 1.5 % agarose gels and visualized by ethidium bromide staining.

**Table 1:** Gene-specific oligonucleotides for sqRT-PCR. Primer sets were designed across exon-exon boundaries to avoid amplification of genomic DNA in the cDNA samples that might contain traces of genomic DNA. ALK1 Primers were also used for qRT-PCR analyses (own assay).

Gene	Forward primer (5' – 3' orientation)	Reverse primer (5' – 3' orientation)	PCR annealing Temp (°C)
*TLS*	TTGAGTCTGTGGCTGATTAC	CACTGGTTGCATTCATTCCT	56
*GADD153*	GTCTAAGGCACTGAGCGTAT	TGGGGAATGACCACTCTGTT	58
*IL-8*	GATTGAGAGTGGACCACACT	TCGGATATTCTCTTGGCCCT	58
*SMAD6*	GTCTTACACTGAAACGGAGG	AGCTGATGCGGACGCTGTTG	58
*COL5A1*	TCGATCCTAACCAAGGATGC	TGTGACGCTTCACCGAAGTC	58
*HEF1*	GAAGAATGGGCCGGAGAGCA	GGGAAATGAAATGGGTCTCA	56
*TLR4*	ACAAAATCCCCGACAACCTC	ATGTAGAACCCGCAAGTCTG	58
*ET-1*	CCTGGACATCATTTGGGTCA	AGGGCTTGCCTTTCAGCTTG	58
*CARP*	ACCGCTATAAGATGATCCGA	AATGAAGCTCTGCTCACCAG	56
*HPTPη*	GGGCACTTTCATTGCCATTG	CATAGCTCCCTTTTCCTGGT	58
*DNAJB1*	ACTACTACCAGACGTTGGGC	CCGCTTGTGGGAGATTTTCG	60
*MGSA*	GGGAATTCACCCCAAGAACA	ATCACAGTGGCTGGCATGTT	58
*c-myc*	AGGCTATTCTGCCCATTTGG	CCACATACAGTCCTGGATGA	58
*ALK1*	GCAACCTGCAGTGTTGCATC	CGGATCTGCTCGTCCAGCAC	60
*GAPDH*	ACCACAGTCCATGCCATCAC	TCCACCACCCTGTTGCTGTA	60

### Relative quantification of gene expression using the comparative method (ΔΔC_T_)

Quantitative real-time PCR (qRT-PCR) was used to measure gene expression levels in HMEC-1, HUVEC and ECRF24 cell lines under various conditions. For gene expression analysis, pre-designed gene-specific primer pairs were selected for 17 target genes and 1 endogenous control from a comprehensive catalogue of pre-designed assays named Assays-on-Demand™ (Applied Biosystems) (see Table 2). All assays were based on TaqMan hydrolysis probes labeled with FAM™ (green fluorescent fluorophore 6-carboxyfluorescein). Assays were performed on an ABI PRISM^® ^7900 HT Sequence Detection System (384-well block module). For the ALK1 construct a second assay utilising SYBR Green I^® ^was used in conjunction with the primer pair designed for the sqRT-PCR detection of ectopic *ALK1 *expression along with a SYBR Green I^® ^assay designed to *GAPDH*. The SYBR Green I^® ^assays were performed on an ABI PRISM^® ^7700 Sequence Detection System (96-well block module).

Once RNA was extracted and reverse transcribed the cDNA was diluted 1 in 50 and assayed in 20 μl reaction volumes. For the assays on demand each reaction comprised of 5 μl cDNA, 10 μl 2x qPCR Master Mix with uracil-N-glycosylase (Eurogentec), 1 μl 20x Primer/Probe Assay mix (Applied Biosystems), the SYBR Green I^® ^assays comprised of 5 μl cDNA, 10 μl 2x SYBR Green I^® ^Master Mix (Applied Biosystems) and 10 pmol each primer. Samples were then amplified on an ABI PRISM^® ^7900 HT or 7700 using the following conditions; 50°C for 2 min, 95°C for 10 min followed by 40 cycles of 95°C for 15 sec and 60°C for 1 min. Samples were assayed in triplicate for each gene, and the mean expression was used during subsequent analysis. Analyses were done with the Applied Biosystems SDS 7900 or 7700 system software, version 2.2 or 1.2, respectively. Relative expression was calculated using the comparative ΔΔC_T _method (Bulletin #2, ABI, Foster City, USA). In brief, the comparative method of analysis compares the amounts of target gene expression relative to an endogenous control, such as *GAPDH*, within a sample to normalise the expression. Within a group of samples, one appropriate sample is elected as a reference sample. Each sample is then compared to the nominated reference sample to give the relative expression of the target gene compared to this reference sample (see Table 3).

**Table 2:** List of genes analysed by qRT-PCR and the corresponding pre-designed gene-specific primer pair assay (ABI Assay-on-Demand™, Applied Biosystems). Where possible RNA specific assays were selected which are depicted by _m1 suffix. For *CHOP *_g1 suffix signifies that the assay may detect DNA and for *ID1 *the _s1 suffix indicates both the primers and probe are in a single exon.

**GENE**	**ABI Assays-on-Demand™**
*ALK1/ACVRL1*	Hs00163543_m1
*ALK5/TGFBR1*	Hs0061319_m1
*BMP6*	Hs00233470_m1
*CARP*	Hs00173317_m1
*CD148/HPTPη*	Hs00174561_m1
*CHOP/DDIT3/GADD153*	Hs00358796_g1
*C-MYC*	Hs00153408_m1
*Collagen VαI/COL5A1*	Hs00609088_m1
*Endoglin/ENG*	Hs00164438_m1
*Endothelin/END1*	Hs00174961_m1
*HEFI/NEDD9*	Hs00610590_m1
*ID1*	Hs00704053_s1
*IL-8*	Hs00174103_m1
*SMAD6/MADH6*	Hs00178579_m1
*TGFβR2*	Hs00559661_m1
*TLR4*	Hs00152939_m1
*GAPDH*	Hs99999905_m1

**Table 3:** The selection of reference samples used for each of the comparative analyses for relative gene expression calculation based on qRT-PCR.

**Reference samples:**	**Test samples:**
HMEC-1 + AdCMV3 24 hr; Experiment 1 HMEC-1 + AdCMV3 24 hr; Experiment 2 HMEC-1 + AdCMV3 24 hr; Experiment 3	HMEC-1 + AdALK1^QD ^24 hr; Experiment 1 HMEC-1 + AdALK1^QD ^24 hr; Experiment 2 HMEC-1 + AdALK1^QD ^24 hr; Experiment 3
HMEC-1 + AdCMV3 4 hr; Experiment 1 HMEC-1 + AdCMV3 4 hr; Experiment 2	HMEC-1 + AdALK1^QD ^4 hr; Experiment 1 HMEC-1 + AdALK1^QD ^4 hr; Experiment 2
HUVEC, no virus	HUVEC + AdCMV3 HUVEC + AdALK1^QD^
ECRF24 cells no virus	ECRF24 + AdLacZ ECRF24 + AdALK1^QD^
HMEC-1 no TGF-β1, 16 hr incubation	HMEC-1, 0.5 ng/ml TGF-β1, 16 hr incubation HMEC-1, 4.0 ng /mlTGF-β1, 16 hr incubation
HMEC-1 no TGF-β1, 24 hr incubation	HMEC-1, 0.5 ng /mlTGF-β1, 24 hr incubation HMEC-1, 4.0 ng/ml TGF-β1, 24 hr incubation

## Results

### Gene expression in HMEC-1 cells 24 hours post AdALK1^QD ^infection

In order to evaluate gene expression specifically induced by ALK1, and not by ALK5, HMEC-1 cells, a human microvascular endothelial cell line [30], were infected with the recombinant adenovirus AdALK1^QD ^expressing the constitutively active form of ALK1. HMEC-1 cells infected with the empty adenovirus vector AdCMV3 served as reference. 24 hours after infection total RNA was isolated and used for hybridization on an Affymetrix HuGeneFL GeneChip™ representing over 5,600 genes. This experiment was produced in triplicate, and only genes that showed at least a two-fold expression change were considered for further evaluation. 11 genes displayed more than a 2-fold increase in all three experiments. 22 genes showed increased expression in two out of three experiments. 15 genes were up-regulated by 2-fold or more in the first two replicate experiments, and 4 genes were up-regulated by ≥ 2-fold in replicate experiments 1 and 3. Three genes were up-regulated by ≥ 2-fold in replicate experiments 2 and 3. We tried to identify genes that were down-regulated by ALK1 signalling but there was no consistent expression pattern of down-regulated genes among the three experiments. Table 4 lists all the genes up-regulated by ≥ 2-fold in at least two out of three experiments. Genes are arranged according to groupings based on current known biological functions.

**Table 4:** Constitutively active ALK1 regulated gene expression in HMEC-1 cells after 24 hours. Gene expression profiling by Affymetrix GeneChip™ and qRT-PCR analysis for HMEC-1 cells (human microvascular endothelial cell line) infected with empty adenovirus (AdCMV3) or AdALK1^QD^. Listed are genes up-regulated in at least two out of three independent experiments. Cells were infected with an MOI of ~50. Gene expression was assesed after 24 hours. Gene expression profile of AdCMV3 infected cells served as reference. GeneChip™ raw data was analysed using Affymetrix MAS 4.0 comparison analysis. Listed are the mean results. Information about gene function was taken from NCBI Entrez Gene data base if not otherwise indicated. qRT-PCR based relative gene expression was calculated using the comparative ΔΔC_T _method. RQ represents the relative expression level (fold change) compared to the reference sample. RQ_Max/Min _are the relative maximum/minimum expression levels of the mean expression level RQ.

**Gene/Gene aliases [GenBank number]**				
**Transcription factors**	**Gene description**	**GeneChip™fold change **(StdDev)	**qRT-PCR RQ **(RQ_Max/Min_)	**TGF-β regulated **(Ref.)

*Inhibitor of DNA binding 1 (ID1) *(represents [S78825])	role in cell growth, senescence, and differentiation, involved in angiogenesis	2.86 (0.71)	6.56 (9.83/4.42)	[6, 70]
*Inhibitor of DNA binding 1B (ID1B) *[S78825]	role in cell growth, senescence, and differentiation, involved in angiogenesis	14*	nt	-
*Inhibitor of DNA binding 2 (ID2) *[M97796]	role in cell growth and differentiation	17.6*	nt	[70]
*Inhibitor of DNA binding 2B (ID2B) *[M96843]	function unknown	8.15*	nt	-
*Ankyrin repeat domain 1 (ANKRD1)/CARP *[X83703]	endothelial and smooth muscle cell expression, angiogenic activity [71]	4.46 (2.80)	4.29 (13.70/2.41)	[41]
*High mobility group AT-hook 2 (HMGA2) *[U28749]	HMG proteins are essential components of the enhancesome, may act as a transcriptional regulating factor	6*	nt	-
**Signalling molecules**				

*Interleukin 8 (IL-8) *[M28130]	inflammatory response, angiogenic factor	18.26 (10.83)	1056.07 (1686.10/666.22)	[72-74]
*Endothelin (EDN1)/ET-1 *[J05008]	vaso constrictor, involved in angiogenesis	3.36 (0.30)	3.87 (5.16/2.97)	[75]
*Chemokine (C-X-C motif) ligand 1 (CXCL1)/GROα1/MGSA *[X54489]	immune response, tumor progression, angiogenic activity	8.2*	nt	[70]
*Bone morphogenetic protein 6 (BMP6) *[M60315]	induced by proinflammatory cytokines, involved in bone and cartilage development	3.8*	4.26 (5.60/3.30)	[70, 76]
*Heme oxygenase-1 (HO-1) *[X06985]	heme catabolism, beneficial in cerebrovascular events, vasodilator	3.8*	nt	[77]
*Jagged 1 (JAG1) *[U73936]	ligand for notch 1, involved in angiogenesis	3.9*	nt	[78]
*Parathyroid hormone-like hormone (PTHLH) *[M24351]	involved in cell proliferation, apoptosis, cancer	2.35*	nt	[79, 80]
**Signal transduction/regulation**				

*SMAD6 *[U59914]	inhibitor of TGF-β family member signaling 2	14.2 (9.41)	14.56 (18.63/11.63)	[81]
*Toll-like receptor 4 (TLR4) *[U93091]	LPS innate immune response	4.33 (1.19)	3.98 (5.51/2.92)	-
*Protein tyrosine phosphatase, receptor type, J (PTPRJ)/CD148/HPTPη *[D37781]	involved in cell proliferation, differentiation, cell motility	3.56 (1.58)	2.08 (3.01/1.47)	-
*SMAD7 *[AF010193]	inhibitor of TGF-β family member signaling 2	7.2*	nt	[81]
*Ras homolog gene family, member B (RHOB) *[M12174]	role in endosomal trafficking [82]	3.25*	nt	[70]
*Mitogen-activated protein kinase kinase kinase 5 (MAP3K5) *[U67156]	activates c-Jun N-terminal kinase (JNK)	3.2*	nt	-
*Endoglin (ENG)/CD105 *(J05481)	TGF-β1, -β3 accessory receptor and signaling modulator	nd	2.36 (3.08/1.84)	[83]
**Extracellular matrix**				

*Collagen 5α1 (COL5A1) *[D90279]	controls the initiation of collagen fibril assembly [62]	7.13 (4.27)	2.28 (2.93/1.81)	[59, 60]
*Serine (or cysteine) proteinase inhibitor 1 (SERPINH1)/HSP47 *[D83174]	assists maturation of the ECM protein type I collagen	2*	nt	[58]
**Stress response**				

*DNA-damage-inducible transcript 3 (DDIT3)/ GADD153 *[S40706]	transcription factor, associated with cell stress and apoptosis	19.5 (2.60)	4.55 (6.42/3.27)	-
DnaJ (Hsp40) homolog, subfamily B, member 1 (DNAJB1) D85429]	linked to the 26S proteasome, up-regulated in bipolar disorder	3.03 (1.00)	nt	-
*Homocysteine-inducible, endoplasmic reticulum stress-inducible, ubiquitin-like domain member 1 (HERPUD1) *[D14695]	linked to the 26S proteasome, up-regulated by Wnt-1	4.1*	nt	-
**Cell adhesion and cytoskelet**				

*Neural precursor cell expressed, developmentally down-regulated 9 (NEDD9)/ HEF1 *[L43821]	actin filament bundle formation, cell adhesion, cytoskeleton organization and biogenesis, integrin mediated signalling pathway, regulation of cell cycle, regulation of cell growth, signal transduction	5.8 (0.95)	3.93 (5.74/2.73)	[84]
*Crystallin, alpha B (CRYAB) *[S45630]	member of the small heat shock protein (HSP20) family, assists assembly of desmin filaments [85]	11.1*	nt	[85, 86]
Tropomyosin 1 (alpha) (TPM1), splice variant 2	associated with the actin filaments	2.3*	nt	[87, 88]
*Poliovirus receptor (PVR)/CD155 *[M24407]	also involved in the regulation of cell adhesion and cell motility [89]	2.35*	nt	-
*Solute carrier family 3, member 2 (SLC3A2)/CD98 *[M21904]	activator of dibasic and neutral amino acid transport, mediates integrin signaling [90]	2.5*	nt	-
**Miscellanous**				

*Leukocyte receptor cluster (LRC) member 4 (LENG4) *[S82470]	Over-expressed in bladder and breast carcinoma	2.1*	nt	-
*Karyopherin alpha 3 (KPNA3) *[Y12394]	nuclear transport protein 91a	2.75*	nt	-
*Ankyrin repeat domain 15 (ANKRD15) *[D79994]	plays a role in cell growth	2.3*	nt	-
*Squalene epoxidase (SQLE) *[D78129)]	catalyzes the first oxygenation step in sterol biosynthesis	2.45*	nt	-

The measurements of fold-change with a ~symbol in front of them are an approximation because the average difference in the reference samples were not above background; *mean of two experiments; nd, not detected; nt, not tested.

Highest induction in all three experiments was seen for a probe with no assigned GenBank number named by Affymetrix as oncogene *TLS/CHOP *suggesting that this probe represents a fusion gene. Other names for *CHOP *are *DDIT3 *or *GADD153*. sqRT-PCR revealed that *GADD153 *was the gene in question and not *TLS *(data not shown). Therefore, all further analyses were done for *GADD153*.

To validate the gene array results we performed qRT-PCR and sqRT-PCR for a subset of genes using the same RNA that was used for the GeneChip™ hybridizations. qRT-PCR was done for genes up-regulated in all three experiments as well as for *BMP6 *(increased expression only seen in experiment 1 and 2), *ALK5*, *TβRII*, *endoglin *and *c-myc*. Our gene array data did not suggest an influence of ALK1 on *endoglin *or *c-myc *expression but previous microarray data had shown that ALK1 signalling up-regulates *endoglin *in primary HUVECs [35] and down-regulates *c-myc *in primary dermal microvascular endothelial cells [36]. Because, TGF-β1 is known to induce its own receptors [37], *ALK5 *and *TβRII *expression was also examined by qRT-PCR. Also tested by sqRT-PCR was the chemokine *MGSA/GROα *that had shown increased expression only in two out of three experiments. qRT-PCR revealed that *endoglin *and *BMP6 *expression was increased by more than 2-fold in all three experiments (Table 4). Up-regulated expression in all three experiments was also seen for *MGSA/GROα *(data not shown). Expression values for *ALK5*, *TβRII *and *c-myc *were unchanged across methods.

The ectopic expression of *ALK1 *due to AdALK1^QD ^infection was not detected by GeneChip™ hybridization in the first two experiments. The ABI qRT-PCR assay for *ALK1 *is designed against exon 1/exon 2 but the coding sequence for *ALK1 *starts in exon 2. Thus, the ABI assay Hs00163543_m1 will not detect *ALK1 *expression from the AdALK1^QD ^construct, since this one contains only the *ALK1 *coding sequence. qRT-PCR assays with primers located in exon 7/exon 8 (own assay) revealed *ALK1 *expression in all three experiments with a mean expression level of RQ = 5079.92 (8659.09/2475.21/4105.47) confirming high levels of ectopic ALK1^QD ^expression.

### Gene expression in HMEC-1 cells 4 hours post AdALK1^QD ^infection

The previous experiments assayed ALK1 induced gene expression after 24 hours. In order to identify ALK1 early response genes, HMEC-1 cells were infected with AdALK1^QD ^and RNA was isolated 4 hours post-infection. AdCMV3 infected HMEC-1 cells served as reference. The isolated RNA of two independent experiments was used for Affymetrix HuGeneFL GeneChip™ analysis as well as for qRT-PCR analysis for a selected set of genes (genes up-regulated after 24 hours in all three experiments). By qRT-PCR analysis AdALK1^QD ^infected cells showed high ectopic *ALK1 *expression (own qRT-PCR assay) with a mean level of RQ = 157.795. Most of the genes identified to be up-regulated after 24 hours showed no significant changes over controls after 4 hours except for *IL-8 *(RQ = 0.595) and *GADD153 *(RQ = 0.69), which were consistently down-regulated. This time dependent response suggests that there is an early response component to ALK1 signalling for *IL-8 *and *GADD153 *but their subsequent up-regulation after 24 hours might be an indirect effect of prolonged ALK1 signalling.

Affymetrix HuGeneFL GeneChip™ analysis identified 13 genes to be up-regulated by more than 1.5-fold and 2 genes were down-regulated by more than 1.5-fold in both experiments (see Table 5). For the purpose of identifying early response genes the threshold for fold changes in gene expression was this time set to 1.5. *ALK1 *expression levels were measured to be 53.85 (experiment 1: 58.5; experiment 2: 49.2). Thus, both the GeneChip™ hybridization and qRT-PCR methods demonstrated high ectopic *ALK1*^QD ^expression levels already after 4 hours of viral infection. Of the genes up-regulated after 24 hours only two genes, *SMAD7 *and *KPNA3*, showed increased expression after 4 hours suggesting these genes were directly regulated by ALK1 signalling. The identified early response genes can be roughly divided into four functional groups: transcription factors (*REST*, *TEAD4 *and *NFIB*), vesicle transport/protein sorting (*RAB6A*, *TM9SF2 *and *PICALM*), targeted protein degradation (*CUL4A*, *COPS6*), and signalling (*KPNA3*, *SMAD7*, *GNA13*, *NF2*, *PLCL1*). *LOC400986 *and *BCAT1 *are classified as miscellanous due to the lack of clear functional data to our knowledge.

**Table 5:** Constitutively active ALK1 regulated gene expression in HMEC-1 cells after 4 hours. Gene expression profiling by Affymetrix GeneChip™ analysis for HMEC-1 cells (human microvascular endothelial cell line) infected with empty adenovirus (AdCMV3) or AdALK1^QD^. Cells were infected with an MOI of ~50. Gene expression was assessed after 4 hours. Gene expression profile of AdCMV3 infected cells served as reference. GeneChip™ raw data was analysed using Affymetrix MAS 4.0 comparison analysis. Listed are the mean results of two independent experiments. Information about gene function was taken from NCBI Entrez Gene data base if not otherwise indicated.

**Gene/Gene aliases [GenBank number]**	**Gene description**	**GeneChip™ fold change**	**TGF-β regulated**
*RAS oncogene family member 6A (RAB6A) *[M28212]	regulates a retrograde transport route connecting endosomes and the endoplasmic reticulum (ER) via the Golgi apparatus	4.35 (3.6/5.1)	-
*LOC400986 *[U28831]	protein immuno-reactive with anti-PTH polyclonal antibodies	4.1 (3.1/5.1)	-
*RE1-silencing transcription factor (REST) *[U22314]	may function as a master negative-regulator of neurogenesis [92]; may play a role in vascular smooth muscle cells and human vascular disease [92, 93]	3.25 (1.8/4.7)	-
*Karyopherin alpha 3 (KPNA3) *[Y12394]	nuclear transport protein [91]	2.9 (3.1/2.7)	-
*Guanine nucleotide binding protein (G protein), alpha 13 (GNA13) *[L22075]	plays a critical role in endothelial cells during vascular development, knock outs are embryonical lethal [54]	2.65 (1.8/3.5)	-
*Transmembrane 9 superfamily member 2 (TM9SF2) *[U81006]	may function as an endosome ion channel or small molecule transporter	2.45 (3.3/1.6)	-
*SMAD7 *[AF010193]	inhibitor of TGF-β family member signaling	2.35 (1.9/2.8)	[81]
*Cullin *4A (CUL4A) U58090]	belongs to the cullin family of E3 ubiquitin ligases	2.3 (2.0/2.6)	-
*Branched-chain aminotransferase 1 (BCAT1) *[U21551]	cytosolic and mitochondrial function, direct genetic target for Myc regulation	2.05 (1.8/2.3)	-
*Phosphatidylinositol-binding clathrin assembly protein (PICALM) *[U45976]	involved in clathrin-mediated endocytosis	1.85 (2.1/1.6)	-
*TEA domain family member 4 (TEAD4) *[U63824])	member of the transcriptional enhancer factor (TEF) family, directs muscle-specific gene expression, induces VEGF expression in endothelial cells [55]	1.8 (1.8/1.8)	-
*Nuclear factor I/B (NFIB) *[U85193]	essential for both lung maturation and brain development [94]	1.75 (1.8/1.7)	-
*COP9 constitutive photo-morphogenic homolog subunit 6 (COPS6) *[U70735]	subunit of COP9 signalosome, involved in the regulation of Cullin-containing ubiquitin E3 ligases [95]	1.7 (1.9/1.5)	-
*Neurofibromin 2 (NF2)*	similar to members of the ERM (ezrin, radixin, moesin) family, links cytoskeletal components with proteins in the cell membrane	-2.8 (1.6/4.0)	-
*Phospholipase C-like 1 (PLCL1) *[D42108]	involved in inositol phospholipid-based intracellular signaling cascade	-2.4 (2.3/2.5)	-

### Gene expression in AdALK1^QD ^infected ECRF24 cells and primary HUVECs

To determine if different endothelial cell types react differently to ALK1 signalling we compared AdALK1^QD^-infected ECRF24 and primary HUVEC cells versus HMEC-1 cells. We used cells infected with AdCMV3 or the same vector containing an irrelevant gene, LacZ (AdLacZ), as controls for bystander effects of viral infection. Non-infected ECRF24 cells and HUVECs served as reference.

Infection with recombinant control adenoviruses had no effect on *IL-8 *or *GADD153*, whereas it appears as if viral infection leads to decreased *HEF1*, *CARP*, *ALK5 *and *ALK1 *expression in HUVECs as well as ECRF24 cells. Constitutively active ALK1 signalling induced different gene expression responses in ECRF24 cells, HUVECs and HMEC-1 cells (see Table 6). ALK1^QD ^up-regulated *GADD153*, *IL-8*, *SMAD6*, and *ID1 *in all three endothelial cell types. Increased expression of *TLR4*, *endothelin*, *CARP *and *endoglin *was only seen for primary HUVECs and HMEC-1 cells but not for ECRF24. In contrast, increased *HEF1 *expression by more than 3-fold was observed for ECRF24 and HMEC-1 cells but not for primary HUVECs. Furthermore, constitutively active ALK1 signalling decreased *TβRII *expression in ECRF24, but not HUVECs or HMEC-1 cells, and ALK5 expression was completely blocked in primary HUVECs, but unchanged in ECRF24 and HMEC-1 cells. These results suggest that within the endothelial cell lineage there is a notable cell type difference in ALK1-induced gene expression.

**Table 6:** Constitutively active ALK1 regulated gene expression in primary HUVECs and the HUVEC cell line ECRF24. ECRF24 cells were infected with an MOI of ~50 with the recombinant adenovirus AdLacZ, containing the LacZ gene, or AdALK1^QD^, respectively. Primary HUVECs were infected with an MOI of ~50 with the empty AdCMV3 adenovirus or AdALK1^QD^, respectively. Gene expression was assessed after 24 hours by qRT-PCR. Non-infected cells served as reference. qRT-PCR based relative gene expression was calculated using the comparative ΔΔC_T _method. RQ represents the relative expression level (fold change) compared to the reference sample. RQ_Max/Min _are the relative maximum/minimum expression levels of the mean expression level RQ. The right column titled HMEC-1 shows in comparison the mean RQ level of three independent experiments with HMEC-1 cells infected with AdALK1^QD ^(see Table 4). Genes with RQ values in bold are considered to be up-regulated.

	**ECRF24**		**primary HUVECs**		**HMEC-1**
**Gene [GenBank number]**	AdLacZ RQ (RQ_Max/Min_)	AdALK1^QD^RQ (RQ_Max/Min_)	AdCMV3 RQ (RQ_Max/Min_)	AdALK1^QD^RQ (RQ_Max/Min_)	AdALK1^QD^RQ

*GADD153 *[S40706]	0.95 (1.07/0.85)	**4.21 (5.27/3.37)**	1.17 (1.27/1.07)	**7.53 (12.05/4.70)**	**4.55**
*IL-8 *[M28130]	1.13 (1.01/1.27)	**11.55 (13.60/9.81)**	1.52 (2.55/0.90)	**4.37 (5.57/3.44)**	**1056.07**
*SMAD6 *[U59914]	0.82 (0.90/0.75)	**5.65 (6.59/4.84)**	1.30 (1.44/1.17)	**38.89 (44.26/34.18)**	**14.56**
*COL5A1 *[D90279]	1.04 (1.15/0.94)	1.68 (1.94/1.45)	1.00 (1.10/0.92)	**3.47 (4.06/2.96)**	**2.28**
*HEF1 *[L43821]	0.89 (0.98/0.81)	**3.88 (4.55/3.31)**	0.61 (0.66/0.56)	1.18 (1.75/0.80)	**3.93**
*TLR4 *[U93091]	0.95 (1.06/0.85)	1.59 (1.82/1.39)	1.02 (1.12/0.93)	**4.77 (5.70/3.99)**	**3.98**
*ET-1 *[J05008]	0.64 (0.70/0.59)	0.73 (0.86/0.61)	1.07 (1.16/0.99)	**2.21 (2.62/1.87)**	**3.87**
*CARP *[X83703]	0.61 (0.74/0.50)	0.79 (1.08/0.58)	0.83 (1.00/0.69)	**1.94 (2.82/1.33)**	**4.29**
*HPTPη *[D37781]	1.69 (1.90/1.51)	1.54 (2.75/0.86)	#	#	**2.08**
*ID1 *[S78825]	0.70 (0.78/0.63)	**2.06 (2.45/1.73)**	1.40 (1.79/1.09)	**4.04 (6.63/2.46)**	**6.56**
*BMP6 *[M60315]	0.64 (0.70/0.57)	0.72 (0.82/0.63)	0.91 (1.15/0.73)	1.61 (2.04/1.27)	**4.26**
*ALK5 *[L11695]	0.68 (0.78/0.59)	0.95 (1.25/0.71)	0.41 (0.44/0.38)	nd	1.36
*TβRII *[M85079]	1.18 (1.31/1.06)	0.77 (0.91/0.65)	0.96 (1.04/0.89)	1.38 (1.65/1.16)	1.26
*Endoglin *[J05481]	0.83 (0.93/0.74)	1.19 (1.60/0.89)	1.02 (1.10/0.95)	**3.05 (4.07/2.29)**	**2.36**
*c-myc *[V00568]	0.85 (1.03/0.70)	0.78 (0.90/0.67)	1.00 (1.17/0.86)	1.05 (1.64/0.67)	1.45
*ALK1 *[L17075] (ABI assay)	0.95 (1.09/0.83)	0.93 (1.11/0.77)	0.86 (0.93/0.79)	1.44 (2.28/0.91)	1.40
*ALK1 *[L17075] (own assay)	0.50	597.72*	0.55	197.18*	5079.92*

nd, not detected after 40 cycles; #, not detected after 40 cycles and not detected in the reference sample; *, ectopic expression

### TGF-β1 regulated gene expression of ALK1 response genes in HMEC-1 cells

Thus far, ALK1 response genes were induced by a constitutively active ALK1 that does not require ligand binding for signal initiation. Since ALK1 is a TGF-β1 receptor, we wanted to know if these genes become regulated by TGF-β1, and in particular whether there are any differences between high or low concentrations of TGF-β1, which reportedly activate either the ALK5 or the ALK1 pathway [6,29].

Approximately 80% confluent cells were incubated with 0.5 ng/ml or 4 ng/ml TGF-β1, respectively, for 16 hours and 24 hours, respectively, which would reflect the time intervall at which ALK1^QD ^regulated gene expression was assesed before. Expression levels of ALK1 response genes were investigated by qRT-PCR and levels in TGF-β1-treated cells were compared to non-TGF-β1 treated cells. The expression profiles of TGF-β1 treated cells after 16 hours or 24 hours were almost identical regardless of TGF-β1 concentration (Table 7, for simplification only the 24 hr expression levels are listed). Gene expression levels with RQ values at or above 2 were considered to be increased and RQ levels below 0.90 were considered to be decreased. Four genes, *COL5A1*, *HEF1*, *endothelin *and *BMP6 *were up-regulated at both concentrations and time points; surprisingly *c-myc *expression was also increased being maximally up-regulated after 16 hours [RQ 1.66 (0.5 ng TGF-β1/ml) and RQ 1.99 (4 ng TGF-β1/ml)]. Three genes, *GADD153*, *IL-8 *and *SMAD6 *previously shown to be up-regulated by constitutively active ALK1 showed suppression at all conditions. *TLR4*, *CARP *and *ID1 *expression after 24 hours was decreased after treatment with low TGF-β1 concentrations but unchanged with high TGF-β1 concentrations. In summary, most of the studied genes showed a distinct expression profile in response to constitutively active ALK1 and TGF-β1 and only *COL5A1*, *HEF1*, *endothelin *and *BMP6 *expression was concordant.

**Table 7:** TGF-β1 regulated gene expression of ALK1 response genes in HMEC-1 cells. HMEC-1 cells (human microvascular endothelial cell line) were incubated with the indicated amounts of TGF-β1. Gene expression after 24 hours was assesed by qRT-PCR. Gene expression profile of non-TGF-β1 treated cells served as reference. qRT-PCR based relative gene expression was calculated using the comparative ΔΔC_T _method. RQ represents the relative expression level (fold change) compared to the reference sample. RQ_Max/Min _are the relative maximum/minimum expression levels of the mean expression level RQ. The right column shows in comparison the average RQ level of three independent experiments with HMEC-1 cells infected with AdALK1^QD ^(see Table 4). Genes with RQ values in bold are considered to be up-regulated and RQ values in italic are considered to be down-regulated.

	**HMEC-1**		
	0.5 ng TGF-β1/ml	4 ng TGF-β1/ml	AdALK1^QD^

**Gene [GenBank number]**	24 hours RQ (RQ_Max/Min_)	24 hours RQ (RQ_Max/Min_)	RQ

*GADD153 *[S40706]	*0.64 (1.55/0.27)*	*0.56 (0.74/0.43)*	**4.55**
*IL-8 *[M28130]	*0.17 (0.32/0.09)*	*0.14 (0.16/0.13)*	**1056.07**
*SMAD6 *[U59914]	*0.74 (0.83/0.66)*	*0.78 (0.87/0.69)*	**14.56**
*COL5A1 *[D90279]	**1.75 (2.00/1.53)**	**2.65 (3.51/2.01)**	**2.28**
*HEF1 *[L43821]	**3.26 (3.50/3.05)**	**3.37 (3.99/2.85)**	**3.93**
*TLR4 *[U93091]	*0.80 (1.05/0.61)*	0.96 (1.07/0.87)	**3.98**
*ET-1 *[J05008]	**3.27 (3.47/3.09)**	**3.06 (3.52/2.66)**	**3.87**
*CARP *[X83703]	*0.89 (1.21/0.65)*	1.32 (2.41/0.72)	**4.29**
*HPTPη *[D37781]	0.94 (1.12/0.79)	1.14 (1.62/0.80)	**2.08**
*ID1 *[S78825]	*0.72 (0.79/0.65)*	0.91 (1.07/0.78)	**6.56**
*BMP6 *[M60315]	**2.44 (2.72/2.19)**	**2.60 (2.82/2.39)**	**4.26**
*ALK5 *[L11695]	1.07 (1.16/0.98)	1.43 (1.64/1.25)	1.36
*TβRII *[M85079]	0.97 (1.10/0.85)	0.99 (1.04/0.94)	1.26
*Endoglin *[J05481]	1.04 (1.22/0.88)	1.22 (1.29/1.16)	**2.36**
*c-myc *[V00568]	1.32 (1.71/1.02)	1.47 (1.88/1.16)	1.45
*ALK1 *[L17075] ABI assay	1.01 (1.23/0.83)	1.23 (1.45/1.04)	1.40
*ALK1 *[L17075] own assay	1.26	1.01	5079.92*

*, ectopic expression

### TGF-β1 dose-dependent regulation of ALK1 response genes in primary HUVECs

Although it was reported that TGF-β1 regulates gene expression differently in a dose dependent manner our analyses for the ALK1 response genes did not reveal such a regulatory mechanism for HMEC-1 cells. However, these experiments were performed with a cell line and immortalized cells may act differently than primary cells as used by Goumans and colleagues [6]. Thus, we used primary HUVECs to investigate what effect different TGF-β1 concentrations might have on the expression of ALK1 response genes. Approximately 80% confluent primary HUVECs were treated with different concentrations of TGF-β1 (0.25 ng/ml, 0.5 ng/ml, 1 ng/ml, 4 ng/ml) for 16 hours. Gene expression levels of a selected set of genes were compared to non-TGF-β1 treated cells by sqRT-PCR.

**Figure 1:**
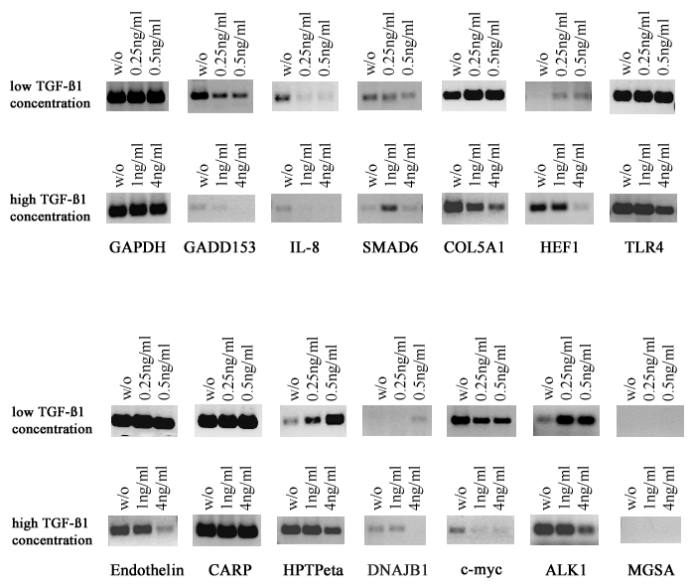
**TGF-β1 regulated gene expression in primary HUVECs. **Primary HUVECs were treated for 16 hours with different TGF-β1 concentrations as indicated. Subsequently, RNA was isolated and gene expression for a set of genes was investigated by semi-quantitative RT-PCR. Expression levels for the *GAPDH *gene served as an internal control.

Several genes showed a clear concentration dependent bi-phasic effect. Low TGF-β1 concentrations (0.25 ng/ml, 0.5 ng/ml) increased the expression of *COL5A1*, *HEF1*, *HPTPη*, *DNAJB1/HSP40 *and *ALK1 *whereas higher concentrations (1 ng/ml, 4 ng/ml) reduced expression, which was most obvious at 4 ng/ml (Figure 1). A second group of genes, *IL-8*, *GADD153 *and *c-myc*, were down-regulated at all TGF-β1 concentrations. For a third group of genes, *TLR4 *and *ET-1*, reduced expression was only observed at 4 ng/ml TGF-β1 and the expression of *CARP *was not changed by any of the TGF-β1 concentrations. *SMAD6 *showed a surprising expression pattern, being up-regulated at the intermediate TGF-β1 concentration of 1 ng/ml but was otherwise unaffected by any other concentration. We also tested for *MGSA *but were unable to detect endogenous expression in HUVECs.

## Discussion

The aim of this study was to identify genes that are regulated by ALK1 signalling in endothelial cells. We were able to identify 34 genes by Affymetrix GeneChip™ and qRT-PCR analysis that were up-regulated in at least two out of three independent experiments in HMEC-1 cells after 24 hours (Table 4) and 15 genes identified by GeneChip™ analysis being regulated after 4 hours (Table 5). Those genes regulated after 4 hours are considered to be early response genes and therefore are most likely direct targets of ALK1 signalling, whereas the genes identified after 24 hours are either late response genes or are indirectly effected by ALK1 signalling. Nevertheless, abrogated or reduced ALK1 signalling would affect the expression of early and late response genes as well as indirectly regulated genes.

HHT is a vascular disorder of localized imbalanced angiogenesis [38] caused by mutations in *ALK1 *or *endoglin*, which implies that ALK1 signalling regulates angiogenesis related genes. Indeed, several of the identified genes in our study are reported to be involved in angiogenesis, tumor angiogenesis and arteriogenesis, i.e. *IL-8 *[39], *ET-1 *[40], *CARP *[41], *HPTPη *[42], *ID1 *[43,44], *MGSA *[45], *SMAD7 *[46], *HO-1 *[47,48], *RHOB *[49], *PTHLH *[50,51], *SERPINH1/HSP47 *[52], *JAG1 *[53], *GNA13 *[54] and *TEAD4 *[55]. Animal studies for some of these genes showed that gene deletion causes embryonic lethality in mice that is accompanied by, or the result of, vascular defects, in part similar to those seen in HHT mice models.

*HPTPη *homozygous mutant mice died before embryonic day 11.5 with vascularisation failure marked by growth retardation, disorganized vascular structures with a notable lack of endoglin expression, an increase in endothelial cell numbers and mitotic activity. Mutant embryos showed homogeneously enlarged primitive vessels defective in vascular remodelling and branching, similar to endoglin-deficient embryos [42]. ID1 activates endothelial cell proliferation and migration, represses the transcription of the angiogenesis inhibitor thrombospondin-1, and *ID1 *expression can be induced by VEGF and TGF-β1 [43]. *RHOB *null mice showed a retarded vascular development characterized by altered sprout morphology. Depletion of RhoB in neonatal rats is associated with apoptosis in the sprouting endothelial cells of newly forming vessels and similarly, in primary endothelial cell culture models RhoB anti-sensense RNA treatment led to apoptosis and failure in tube formation [49]. *Jagged1 *knock-out mice are embryonically lethal with vascular remodelling defects [56]. Mutations in *Jagged1 *cause the Alagille syndrome (AGS) a dominantly inherited multi-system disorder. Intracranial bleeding is a recognized complication and a cause of mortality in AGS due to intracranial vessel abnormalities. There is a body of evidence supporting a role for Jagged1 and the Notch signalling pathway in vascular development, i.e. arterial-venous differentiation [57]. TGF-β1 regulates the expression of extracellular matrix (ECM) proteins.

We identified two genes, *SERPINH1/HSP47 *and *COL5A1*, to be up-regulated by ALK1 that can be directly linked to the ECM. The heat shock protein hsp47 is involved in the maturation of the ECM protein type I collagen, both being induced by TGF-β1 [58]. *COL5A1 *expression in osteoblasts and vascular smooth muscle cells is induced by TGF-β1 [59,60] and haplo-insufficiency leads to the heritable connective-tissue disorder Ehlers-Danlos syndrome that is characterized by an altered collagen-fibrillar structure of the dermis, joints, eyes and blood vessels [61]. *COL5A1 *-/- mice are embryonially lethal around day E10 and death is associated with pooling of blood in the yolk sac, suggesting an insufficient wall integrity to maintain a functioning yolk sac [62]. Mis-expression of Smad7 gave rise to dilated large vessels in the developing chick limb and head, and development of intra and intervascular shunts were frequently observed [46]. It is noteworthy that the ALK1 early response gene *TEAD4/RTEF-1 *is a transcription factor that stimulates VEGF expression and thus promotes endothelial migration and tube formation [54].

The cumulative developmental and biological data for the above described proteins and the fact that their genes are ALK1-regulated suggests that ALK1 is part of an inter-dependent network in vascular homeostasis. This might explain the vascular defects observed in HHT patients and HHT mice models. Many of these genes may be potential candidates in the search for HHT modifier genes. Thus, it would be important now to investigate the expression profile of these genes in the HHT endoglin and ALK1 mice models as well as in HHT patients. Furthermore, the ALK1-response genes *endothelin (ET-1)*, *heme oxygenase (HO-1) *and *BMP6 *might also be involved in other vascular disorders. For example, patients with ALK1 mutations were reported with overlapping symptoms for HHT and primary pulmonary hypertension [63] a disorder effecting endothelial as well as smooth muscle cells. Endothelin and heme oxygenase play a role in vascular tone regulation [64,65] and BMP6 is involved in vascular smooth muscle cell differentiation and being an antagonist of TGF-β1 [66].

ALK1 is a TGF-β1 receptor. We therefore performed a literature search to see which one of the ALK1 regulated genes were reported to be TGF-β1 response genes. We found reports for 19 genes to be regulated by TGF-β1 (Table 4 and 5). Furthermore, our sqRT-PCR results for primary HUVECs (Figure 1) demonstrate that *GADD153*, *HPTPη*, *TLR4 *and *DNAJB1 *expression is also regulated by TGF-β1 adding these genes to the list of TGF-β1 response genes while the remaining genes might be new target genes of TGF-β s or BMPs. In addition, expression of several ALK1-response genes in primary HUVECs are regulated by TGF-β1 in a concentration dependent bi-phasic way (Figure 1), being up-regulated at lower TGF-β1 concentrations and down-regulated at high concentrations. Such a TGF-β dose dependent response mechanism for primary endothelial cells was reported previously [6], showing that low TGF-β1 concentrations (0.25 ng/ml and 0.5 ng/ml) phosphorylated Smad1/5 and enhanced proliferation and migration mediated by ALK1, whereas high TGF-β1 concentrations (≥ 2.5 ng/ml) had the opposite effect mediated by ALK5 due to phosphorylation of Smad2/3. Further experiments are currently under way to investigate how ALK1 might be involved in the bi-phasic activity of TGF-β1 in regulating the here identified ALK1-response genes. In addition, we show to our knowledge for the first time, that *ALK1 *expression in primary HUVECs is regulated by TGF-β1 (Figure 1).

We observed distinct gene expression responses upon ALK1^QD ^signalling among three different endothelial cell types (Table 6, summarized in Table 8), two established endothelial cell lines (HMEC-1, ECRF24) and primary endothelial cells (HUVECs). Cell type differences within the endothelial lineage were also observed for TGF-β1 induced gene expression in primary HUVECs and HMEC-1 cells (summarized in Table 8). TGF-β1 regulated gene expression in HMEC-1 cells did not follow the expression pattern as seen for several genes in primary HUVECs. For example, *COL5A1 *and *HEF1 *were up-regulated in HMEC-1 cells at low and high TGF-β1 concentrations, but in HUVECs these genes followed a dose dependent expression pattern. This difference between different endothelial cell types might explain why there is only minimal overlap between our list of ALK1 regulated genes and previously published reports.

**Table 8:** Overview of constitutively active ALK1 and TGF-β1 regulated gene expression in different endothelial cell types.

	ALK1^QD ^regulated gene expression			low TGF-β1 conc. (≤ 0.5 ng/ml)		high TGF-β1 conc. (≥ 1 ng/ml)	
Gene	**HMEC-1**	**HUVEC**	**ECRF24**	**HMEC-1**	**HUVEC**	**HMEC-1**	**HUVEC**

*GADD153*	**up**	**up**	**up**	***down***	***down***	***down***	***down***
*IL-8*	**up**	**up**	**up**	***down***	***down***	***down***	***down***
*SMAD6*	**up**	**up**	**up**	***down***	**-**	***down***	**up**
*COL5A1*	**up**	**up**	**-**	**up**	**up**	**up**	***down***
*HEF1*	**up**	**-**	**up**	**up**	**up**	**up**	***down***
*TLR4*	**up**	**up**	**-**	***down***	**-**	**-**	***down***
*ET-1*	**up**	**up**	**-**	**up**	**-**	**up**	***down***
*CARP*	**up**	**up**	**-**	***down***	**-**	-	**-**
*HPTPη*	**up**	**-**	**-**	**-**	**up**	-	***down***
*ID1*	**up**	**up**	**up**	***down***	nt	-	nt
*BMP6*	**up**	**-**	-	**up**	nt	**up**	nt
*ALK5*	**-**	***down***	-	**-**	nt	-	nt
*TβRII*	**-**	**-**	***down***	***down***	nt	-	nt
*Endoglin*	**up**	**up**	**-**	**-**	nt	-	nt
*c-myc*	**-**	**-**	**-**	**up**	***down***	**up**	***down***
*ALK1*	**-**	**-**	**-**	**-**	**up**	**-**	***down***

up, up-regulated expression; down, down-regulated expression; -, unchanged expression; nt, not tested

Lamouille and colleagues analysed ALK1 induced gene expression of AdALK1^QD ^infected primary dermal microvascular endothelial cells after 15 hours [30], but there is no overlap regarding the expression profile with our study, and this was despite the HMEC-1 cell line being derived from dermal microvascular endothelial cells. In a study by Ota *et al*. [35], primary HUVECs were used to establish an ALK1 regulated expression profile 48 hours after AdALK1^QD ^infection and only the increased expression for *SMAD6*, *SMAD7*, *ID1*, *ID2 *and *endoglin *agrees with our study. The greatest agreement can be found with a recent analysis by Wu *et al*. [67]. Here, AdALK1^QD ^infected human lung microvascular endothelial cells were used to investigate ALK1 regulated gene expression comparable to the expression profile initiated after 4 hour TGF-β1 treatment as stated by the authors. The authors report increased expression for *GADD153*, *HEF1*, *DNAJB1*, *HO-1*, *SMAD7*, *CRYAB*, *HERPUD1*, *LENG4 *and *SERPINH1*, like in our study but they did not find increased expression for *endoglin*, *SMAD6 *or *ID1 *or *ID2 *as we and Ota *et al*. observed. However, several other genes including *TLR4*, *endoglin*, *JAG1*, *CD155 *and *KPNA3 *which we found to be increased by ALK1 were instead induced by TGF-β1 in the study by Wu *et al*. Furthermore, they showed that the profiles of genes regulated by TGF-β1, ALK1 and ALK5 in lung microvascular endothelial cells are distinct from each other, although some genes are regulated by all of them. This is similar to our data in that there is a difference in TGF-β induced signalling and constitutively active ALK1 signalling in HMEC-1 cells (Table 7, summarized in Table 8) and HUVECs. In HUVECs, constitutively active ALK1 increased *IL-8*, *GADD153 *and *CARP *expression that were either repressed by TGF-β1 or unchanged (Figure 1). How can this be explained? The observed disparities might be due to ligand mediated transient responses versus the sustained responses owing to a constitutively active receptor. Signal intensity and duration are important factors in signal specificity. It is also possible that cell type dependent TGF-β1 acts through Smad-dependent and Smad-independent pathways. Nevertheless, it can not be excluded that further ALK1 ligands like TGF-β3 or may be BMP9 [68] exist that initiate a different gene profile than TGF-β1. In addition, it is also possible that ligand independent ALK1^QD ^regulated gene expression in endothelial cells might partly reflect ALK1-specific responses (i.e. *REST*, *NFIB*, *TEAD4*, *NF2*, *HEF1*) for other cell types like smooth muscle cells or neuronal cells [69].

## Conclusion

In conclusion, we have identified new ALK1 as well as TGF-β1 response genes. Several of those are involved in the different phases of angiogenesis. Being down-stream of the ALK1 signalling pathway the expression of these genes would be affected by ALK1 mutations suggesting that these genes might be involved in the development of HHT. In addition, we showed that ligand induced receptor signalling and subsequent gene expression can be different from gene expression induced by constitutively active receptor signalling. Therefore, the gene expression results obtained from studies with constitutively active receptors like ALK1^QD ^help to identify new target genes but the results may not necessarily reflect the ligand activated gene expression profile. This needs to be established in further assays.

## Abbreviations

ALK, activin receptor-like kinase; BMP, bone morphogenetic protein; conc., concentration; FCS, fetal calf serum; HHT, hereditary hemorrhagic telangiectasia; HMEC, human microvascular endothelial cell; HUVEC, human umbilical vein endothelial cell; MOI, multiplicity of infection; qRT-PCR, quantitative real-time-polymerase chain reaction; RT, reverse transcription; sqRT-PCR, semi quantitative RT-PCR; TGF, transforming growth factor.

## Competing interests

The author(s) declare that they have no competing interests.

## Authors' contributions

AL conceived, designed, coordinated and participated in the experiments of the study and drafted the manuscript. FS carried out the qRT-PCR and statistical analysis, HKD carried out the Affymetrix GeneChip™ experiments and statistical analysis, GK-L generated the recombinant Adenoviruses, MH participated in the coordination of the study and helped to draft the manuscript, PJRD participated in the coordination of the qRT-PCR and helped to draft the manuscript, DAM participated in the design and coordination of the study, JG participated in the design and coordination of the study and helped to draft the manuscript. All authors read and approved the final manuscript.

## Pre-publication history

The pre-publication history for this paper can be accessed here:


